# Gene expression changes in long-term culture of T-cell clones: genomic effects of chronic antigenic stress in aging and immunosenescence

**DOI:** 10.1111/j.1474-9726.2007.00269.x

**Published:** 2007-04-01

**Authors:** Dawn J Mazzatti, Andrew White, Rosalyn J Forsey, Jonathan R Powell, Graham Pawelec

**Affiliations:** 1 Unilever Corporate Research Colworth Park, Sharnbrook, Bedford MK44 1LQ, UK; 2 LCG Biosciences Bourn, Cambridge CB3 7TR, UK; 3 Zentrum für Medizinische Forschung, Universitätsklinikum Tübingen Waldhörnlestr. 22, D-72072 Tübingen, Germany

**Keywords:** aging, immunosenescence, microarray, T-cell clone

## Abstract

The adaptive immune response requires waves of T-cell clonal expansion on contact with altered self and contraction after elimination of antigen. In the case of persisting antigen, as occurs for example in cytomegalovirus or Epstein–Barr virus infection, this critical process can become dysregulated and responding T-cells enter into a dysfunctional senescent state. Longitudinal studies suggest that the presence of increased numbers of such T-cells is a poor prognostic factor for survival in the very elderly. Understanding the nature of the defects in these T-cells might facilitate intervention to improve immunity in the elderly. The process of clonal expansion under chronic antigenic stress can be modelled *in vitro* using continuously cultured T-cells. Here, we have used cDNA array technology to investigate differences in gene expression in a set of five different T-cell clones at early, middle and late passage in culture. Differentially expressed genes were confirmed by real-time polymerase chain reaction, and relationships between these assessed using Ingenuity Systems evidence-based association analysis. Several genes and chemokines related to induction of apoptosis and signal transduction pathways regulated by transforming growth factor β (TGFβ), epidermal growth factor (EGF), fos and β-catenin were altered in late compared to early passage cells. These pathways and affected genes may play a significant role in driving the cellular senescent phenotype and warrant further investigation as potential biomarkers of aging and senescence. These genes may additionally provide targets for intervention.

## Introduction

The frequency of näive T-cells carrying receptors for a particular antigen is very low, so that in response to challenge, T-cell clones (TCC) must rapidly undergo numerous rounds of cell division in order to produce sufficient cells to cope with the antigenic insult. From work on infectious mononucleosis, it was estimated that about 28 population doublings were required for resolution of acute disease (Maini *et al.*, 1999) agreeing remarkably well with estimates made on vaccinating cancer patients (Boon *et al.*, 2006). Excess cells are eliminated by apoptosis at disease resolution, with retention of memory cells possibly at a 1-log higher frequency than the original naïve cells (Boon *et al.*, 2006). However, in chronic disease, antigenic stimulation continues and the responding TCCs may become ‘exhausted’ (Zinkernagel *et al.*, 1999). In humans, cytomegalovirus (CMV) contributes markedly to the persistent clonal expansions commonly seen in the elderly (Khan *et al.*, 2002; Ouyang *et al.*, 2003; Hadrup *et al.*, 2006). Moreover, the absolute number of accumulated cells is an important part of the ‘immune risk profile’ (IRP) predicting mortality in longitudinal studies of the very elderly (Wikby *et al.*, 2005). Strikingly, it is not just the number of cells, but the number of different expanded clones, which is also associated with the IRP (Hadrup *et al.*, 2006). These accumulated cells are dysfunctional, and may not only be filling the available ‘immunological space’ but may also be actively suppressive of responses of other clones (Pawelec *et al.*, 2006a). Learning more about the reasons for accumulation of such dysfunctional cells may therefore also bring practical benefits in immune intervention in the elderly to reconstitute appropriate immune responses.

Studies of T-cell clonal behaviour under chronic antigenic stress in culture may assist in this endeavour and also offer the opportunity of testing interventions *in vitro*. To this end, we have investigated changes in TCCs over their finite lifespans in culture, as a model of T-cell immunosenescence (Pawelec *et al.*, 1997). Both the average and maximal lifespans of human TCCs in culture, their culture passage-associated changes in surface molecule expression, and their altered cytokine secretion patterns (Pawelec *et al.*, 2006b), appear to mirror quite faithfully what we believe happens *in vivo* under chronic antigenic stress (Pawelec *et al.*, 2006a). Here, cDNA array technology is employed for the first time on TCCs in an effort to determine age-associated changes in gene expression that may contribute to the senescent phenotype.

## Results

In order to examine the effect of *in vitro* culture-driven senescence on gene expression profiling, RNA samples from TCCs were isolated at various time points for microarray analysis (Table 1a). Gene expression profiling of early passage, presenescent TCCs compared to late passage, senescent clones demonstrated that 43 genes were differentially expressed in T-cell immunosenescence. Of these, 30 were upregulated and 13 downregulated (Table 2). To confirm that these genes identified through microarray analysis were truly differentially expressed, stringent data analysis was performed. After exclusion of genes with missing spots, spots of poor quality, genes that did not dye swap, and genes below detection threshold, gene profiling was performed over the lifespan of each individual clone. Only genes with a differential expression of at least threefold in three out of five clones, and which passed analysis of variance (anova) testing were considered to be truly differentially expressed.

**Table 1a:** Experimental samples used for microarray analysis

Donor	Clone	Time in culture (day)	Population doublings
OCTO 433	6	56	29.5
OCTO 433	6	84	42.25
OCTO 433	6	106	50
OCTO 433	7	43	26
OCTO 433	7	85	43.25
OCTO 433	7	115	51
OCTO 433	14	71	38.5
OCTO 433	14	85	46.25
OCTO 433	14	99	50.75
OCTO 433	14	129	55.75
OCTO 433	21	78	41.25
OCTO 433	21	85	44.25
OCTO 433	21	99	49.25
OCTO 433	21	122	54.5
OCTO 433	26	56	32.75
OCTO 433	26	84	46.25
OCTO 433	26	98	52.75
OCTO 433	26	143	66.25

**Table 2:** Genes differentially expressed larger than threefold up or down in T-cell clone (TCC) senescence as assessed by microarray analysis

Gene name	Trend in senescence
Small inducible cytokine subfamily B (Cys-X-Cys), member 10	Increased
Granulysin (GNLY), transcript variant 519	Increased
CCL17 chemokine (C-C motif) ligand 17	Increased
β-thromboglobulin-like protein (IL-8)	Increased
Interferon, α-inducible protein (clone IFI-6-16)	Increased
Adenosine A2a receptor	Increased
PTGS2 prostaglandin-endoperoxide synthase 2 (prostaglandin G/H synthase and cyclooxygenase)	Increased
Gap junction protein	Increased
CD5 antigen-like (scavenger receptor cysteine-rich family)	Increased
CD7 antigen (p41)	Increased
Interferon-induced cellular resistance mediator protein (MxB)	Increased
Neutrophil cytosolic factor 1 (47 kDa, chronic granulomatous disease, autosomal 1)	Increased
Early growth response 2	Increased
Small inducible cytokine A2	Increased
Small inducible cytokine A3	Increased
Ferredoxin reductase	Increased
Relaxin 2 (H2)	Increased
Mitogen-induced nuclear orphan receptor (MINOR)	Increased
Zinc finger protein, subfamily 1A, 3	Increased
Diphtheria toxin receptor (heparin-binding epidermal growth factor-like growth factor)	Increased
Lymphotoxin α (TNF superfamily, member 1)	Increased
Lymphotoxin β (TNF superfamily, member 3)	Increased
FLJ22635 fis	Increased
Tumor necrosis factor receptor superfamily, member 5	Increased
Chemokine (C-C motif) receptor 7 (CCR7)	Increased
2′,5′-oligoadenylate synthetase 1	Increased
Aldolase C	Decreased
Human pregnancy-specific glycoprotein β-1 (SP1)	Decreased
Small inducible cytokine A4	Decreased
Phosphoglycerate kinase 1	Decreased
Early growth response 2	Decreased
Δ-6 fatty acid desaturase	Decreased
NADH dehydrogenase (ubiquinone) 1 β subcomplex	Decreased
Nuclear receptor subfamily 4, group A, member 2	Decreased
Vacuolar protein sorting 45B	Decreased
Insulin upstream factor 1	Decreased
Δ-5 fatty acid desaturase	Decreased
Syntrophin 5; γ2-syntrophin	Decreased
Selectin L (lymphocyte adhesion molecule 1)	Decreased

To validate microarray results and extend the analysis to a larger set of TCCs derived from different octogenarian donors (Table 1b), we arbitrarily selected 20 genes and here show their expression quantified by real-time reverse transcription-polymerase chain reaction (RT-PCR) in nine TCCs at early and late passage. A list of the probe sets used for RT-PCRs is shown in Table 3. We found that both methods detected similar patterns of expression for the 17 upregulated and three downregulated genes. Primary cellular functions of these genes are listed in Table 4.

**Table 1b:** Experimental samples used for semiquantitative real-time reverse transcription-polymerase chain reaction (RT-PCR) analysis

Donor	Clone	Time in culture (day)	Population doublings
OCTO 433	9	43	27
OCTO 433	9	64	32.75
OCTO 433	9	71	41
OCTO 433	9	85	48.75
OCTO 433	9	115	54.75
OCTO 433	14	43	25.5
OCTO 433	14	71	38.5
OCTO 433	14	78	41.75
OCTO 433	14	99	50.75
OCTO 433	25	36	23.75
OCTO 433	25	57	31.75
OCTO 433	25	85	42.5
OCTO 433	25	136	55.5
OCTO 433	26	43	26.75
OCTO 433	26	56	32.75
OCTO 433	26	98	52.75
OCTO 433	26	129	61.25
OCTO 433	26	143	66.25
OCTO 432	4	31	23
OCTO 432	4	45	29.75
OCTO 432	4	52	33.5
OCTO 432	4	80	39.75
OCTO 432	4	94	43.5
OCTO 434	3	30	22.25
OCTO 434	3	50	28.25
OCTO 434	3	64	33.75
OCTO 434	3	94	42.5

**Table 3:** TaqMan (Applied Biosystems) probes used for real-time reverse transcription-polymerase chain reaction (RT-PCR)

Gene	Probe reference
GJB1	Hs00702141
GNLY	Hs00246266
IL-8	Hs00174103
MINOR (NR4A3)	Hs00175077
CCL2	Hs00234140
CCL3	Hs00234142
ALDOC	Hs00193059
ADORA2A	Hs00169123
HBEGF	Hs00181813
LTB	Hs00242737
RLN2	Hs00366471
PTPN13	Hs00196632
CD7	Hs00196191
EGR2	Hs00166165
CXCL10	Hs00171042
TNFRSF5	Hs01002912
SELL	Hs00174151
CD5L	Hs00234667
CCL17	Hs00171074
CCR7	Hs00171054
GAPDH	Hs99999905

**Table 4:** Main cellular functions of genes assessed by real-time reverse transcription-polymerase chain reaction (RT-PCR)

Gene	Full name	Function
GJB1	Gap junction protein	Member of the connexion family that assembles to form gap junction channels that facilitate transfer of ions between cells
GNLY	Granulysin	Protein present in cytotoxic granules of cytotoxic T lymphocytes and NK cells
IL-8	Interleukin 8	Cytokine involved in chemoattraction, angiogenesis, and activation of neutrophils
NR4A3	Mitogen-induced nuclear orphan receptor	Member of the steroid-thyroid hormone-retinoid receptor superfamily
CCL2	Small inducible cytokine A2	Cytokine involved in immunoregulatory and inflammatory processes
CCL3	Small inducible cytokine A3	Macrophage inflammatory protein-1 involved in the acute inflammatory state in recruitment and activation of polymorphonuclear leukocytes
ALDOC	Aldolase C	Glycolytic enzyme that catalyzes the cleavage of fructose-1,6-biphosphate and fructose-1-phosphate
ADORA2A	Adenosine A2A receptor	G-protein coupled receptor family member which activates adenylyl cyclase
HBEGF	Heparin-binding epidermal growth factor-like growth factor	Plays a role in proliferation and differentiation
LTB	Lymphotoxin β receptor	Plays a role in the development and organization of lymphoid tissue
RLN2	Relaxin 2 (H2)	Endocrine and autocrine/paracrine hormone that belongs to insulin gene superfamily
CD7	CD7 antigen	Member of the immunoglobulin superfamily involved in T-cell interactions and lymphoid development
EGR2	Early growth response 2	Transcription factor. Mutations associated with Charcot-Marie-Tooth disease Type 1
CXCL10	Small inducible cytokine (Cys-X-Cys) member 10	Interferon-γ-induced protein that binds CXCR3 causing stimulation of monocytes, NK and T-cell migration
TNFRSF5	Tumor necrosis factor receptor superfamily, member 5 (CD40)	Essential in mediating a broad variety of immune and inflammatory responses including T-cell-dependent immunoglobulin class switching, memory B cell development, and germinal centre formation
SELL	l-selectin	Member of family of adhesion/homing receptors involved in leukocyte-endothelium interactions
CD5L	CD5-antigen-like	
CCL17	CCL17 chemokine (C-C motif) ligand 17	Involved in T-cell development and trafficking and activation of mature T cells
CCR7	Chemokine (C-C motif) receptor 7	Involved in chemotaxis and migration dendritic cells
GAPDH	Glyceraldehyde-3-phosphate dehydrogenase	Housekeeper

**Fig. 1:**
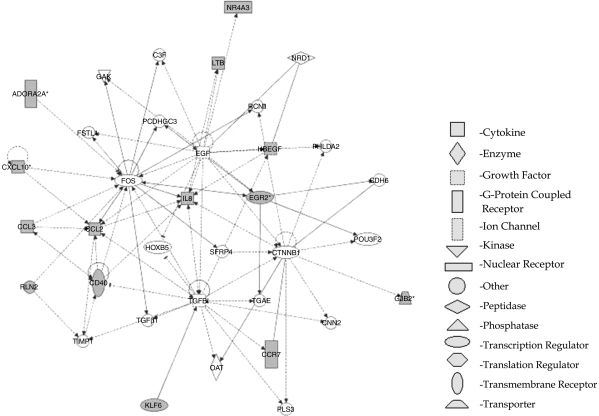
Network of genes differentially expressed in T-cell clone (TCC) immunosenescence. Forty-three genes that were differentially regulated in late passage, senescent, TCCs were analysed by the Ingenuity Pathway Analysis (IPA) tool. The network shown was significantly associated with cellular movement, haematological system development and function, immune response, cell-to-cell signaling and interaction, and cellular growth and proliferation (*P <* 0.01). Shaded genes were identified by microarray analysis as differentially expressed in TCC senescence. Other nodal genes are directly or indirectly associated with the differentially expressed genes. The meaning of the nodal shapes is indicated.

In order to gain an understanding of which functional cellular processes may be most affected during immunosenescence, genes that were significantly altered at TCC senescence were visualized by Ingenuity Pathway Analysis (IPA). Ingenuity entry tool systematically encodes findings presented in peer-reviewed scientific publications into ontologies, or groups of genes/proteins related by common function. A molecular network of direct physical, transcriptional, and enzymatic interactions was computed from this knowledge base. The resulting network contains molecular relationships with a high degree of connectivity and every gene in the network is supported by published information. Genes identified through microarray analysis (focus genes), network score (probability of network being assembled by chance alone), and main cellular functions of the network are listed in Table 5. The one statistically significant molecular network assembled using IPA profiling of genes differentially expressed in immunosenescence, including interacting partners, is depicted in Fig. 1. Genes demonstrated to be differentially expressed following microarray analysis of TCC senescence are coloured in grey. Class of function for each of the genes, including enzymes, growth factors, and receptors, are depicted by nodal shape, as identified in the figure legend.

**Table 5:** Genetic network altered in T-cell senescence

Genes	Score	Focus genes	Top functions
ADORA2A*, C3F, **CCL2**, **CCL3**, **CCR7**, **CD40**, CDH6, CNN2, CTNNA1, CXCL10*, EGF, EGR2*, FOS, FSTC1, GAK, **GJB2**, **HBEGF**, HOXB5, IL-8*, ITGAE, **KLF6**, **LTB**, **NR4A3**, NRD1, OAT, PLDHGC3, PHLDA2, PLS3, POU3F2, RCN1, **RLN2**, SRFP4, TGFBA, TGFBI, TIMP1	33	14	Cellular movement; Haematological system development and function; Immune response

Focus genes are shown in bold. Asterisks denote genes involved in multiple pathways.

Bold genes are those identified by microarray analysis.

Other genes listed were either not on the arrays or were not significantly regulated.

A score of > 3 was considered statistically significant (*P <* 0.001).

## Discussion

Clonal expansion under conditions of chronic antigenic stress is thought to contribute materially to the altered immune status in the elderly. The main source of antigen appears to be derived from CMV, a β herpes virus that persists life long after infection, usually in adolescence in the majority of people. The accumulation of large numbers of dysfunctional CMV-specific cells is an important parameter associated with the ‘IRP’, which predicts mortality in free-living elderly people (Pawelec *et al.*, 2005). Understanding this phenomenon of altered T-cell behaviour associated with ‘exhaustion’ under chronic antigenic stress is essential for developing rational approaches to remediation. Here, we have employed an *in vitro* model of chronic antigenic stimulation at the clonal level to examine alterations in gene expression patterns as T-cells progress through their finite lifespans towards immunosenescence. Therefore, the purpose of the present study was to investigate which genes and signalling pathways were differentially expressed in immunosenescence. For this purpose, we performed gene expression profiling of representative TCCs isolated from a healthy octogenarian. In our data set, all genes selected for validation demonstrated similar results for the microarrays and real-time RT-PCR measurements, yet with different magnitudes. After validation of the microarray results, we looked for groups of aberrantly expressed genes functionally connected through common signalling pathways. Ingenuity pathway analysis identified one network with high probable significance to the regulation of immunosenescence. RNA levels of 14 out of 36 genes in this network were differentially expressed in TCC immunosenescence. Of note, 9 of the other 22 genes constructed in the network were not spotted on the microarray. Of the remaining 13 genes that were spotted on the microarray but not identified as significantly altered in TCC senescence, genes were disregarded for several reasons, including genes that did not dye swap (one gene), spots of poor quality (one gene), genes expressed below the detection threshold (three genes), genes that exhibited no trend in senescence or that were altered in less than three out of five clones investigated (seven genes), and genes that were differentially expressed in senescence but that did not pass anova testing (one gene).

Although the trend in expression pattern of genes identified through microarray analysis was confirmed by RT-PCR for each gene investigated, the magnitude of expression differed between the two methods. Furthermore, the magnitude of RNA expression varied between clones. This may be partially explained by the fact that cells taken from the different clones had been frozen down at different stages in the culture of each clone. Furthermore, the first point of reference was obtained when the individual clone attained > 1 million cells. Therefore, it remains possible that the starting/reference points for each clone were slightly different based on the rate of replication of each clone. This would provide one possible explanation as to why the gene changes that occur in senescence were not observed in all clones. However, clonal heterogeneity not reflected in the homogenous pattern of expression of surface molecules may also have contributed to these differences.

One hallmark of cellular aging is the decreased proliferative response resulting from age-related defects in signal transduction in response to T-cell receptor (TCR)-mediated stimulation (Pawelec *et al.*, 2001). One of the main transcriptional regulators of cellular proliferation is activator protein-1 (AP-1). AP-1 is a family of transcription factors that bind specific DNA sequences in the promoter region of target genes. Fos is part of the heterodimeric AP-1 transcription factor complex which has been implicated in cellular proliferation, differentiation, inflammation and the stress response (Kerppola & Curran, 1995). Many of the genes found to be differentially expressed in TCC senescence – including the adenosine 2A receptor, CXCL10, CCL3, CCL2, IL-8, and relaxin 2 – directly influence fos-mediated gene regulation. As such, fos sits at the centre of the molecular pathway that was identified by IPA (Fig. 1). In fact, regulation of c-fos has been described to be altered in cellular aging concomitant with loss of AP-1 activity (Riabowol *et al.*, 1992; Wheaton *et al.*, 1996; Medicherla *et al.*, 2001; Sheerin *et al.*, 2001). It can be hypothesized that differential expression of fos-regulatory genes may impact on the ability of fos to form the AP-1 complex or may affect concomitant transcriptional activation of AP-1 target genes.

AP-1 transcriptional activity is also regulated by epidermal growth factor (EGF). It has previously been reported that EGF-induced stimulation of AP-1 activity is decreased in human keratinocytes that were cultured *in vitro* until senescence (Shi & Isseroff, 2005). The model of TCC senescence we have used is very similar to this *in vitro* system. In fact, several of the genes that were found to be differentially expressed in TCC senescence – including EGR2, HBEGF, IL-1, LTB, and NR4A3 – can be modulated by EGF activity (Fig. 1). Although we have not measured EGF activity in the current study, it is probable that EGF function would be impaired in our *in vitro* model of senescence, in accordance with previous investigations of aging and senescent cells. This model would provide a mechanism for the alterations in EGF-regulated genes we observe.

One major function of EGF in cellular proliferation is its transcriptional regulation of factors involved in cell-cycle progression. Epidermal growth factor has been demonstrated to stimulate T-cell factor (TCF)/LEF transcriptional activity which is necessary for cell-cycle progression of normal cells (Graham & Asthagiri, 2004). Furthermore, regulation of EGF/TCF-mediated proliferative control is dependent on interaction with β-catenin (CTNNB1). β-catenin is an intracellular protein whose functions range from stabilization of cell–cell adhesion to control over gene expression. β-catenin is tightly regulated by cytosolic degradation and sequestration to the plasma membrane and translocation to the nucleus is required for regulation of target genes including TCF following activation by signalling events (Polakis, 1999; Provost & Rimm, 1999). Ingenuity pathway analysis of senescent TCCs identified several β-catenin-regulatory genes and transcriptional targets of β-catenin, including gap junction B2, CCR7, HBEGF, and IL-8. Altered β-catenin expression and activity has also been described in a variety of cancer types (Korinek *et al.*, 1997; Satoh *et al.*, 2000; Persad *et al.*, 2001). To date, a role for β-catenin in aging or senescence has not been described. Our results suggest that β-catenin activity may be altered in senescent TCCs, but further investigation is necessary to test this hypothesis.

Following acute disease resolution, excess TCCs, with the exception of memory cells, are eliminated by activation-induced cell death (AICD). However, in chronic disease and aging, antigenic stimulation continues and the responding TCCs may become exhausted, leading to changes in their susceptibility to apoptosis (Koch *et al.*, 2006). The data presented in this report indicate that various mediators of apoptosis are upregulated in late-passage TCCs. The finding that PTPN13 (small inducible cytokine B) is upregulated is important as this protein was found to interact with and dephosphorylate the Fas receptor and IkB-α, suggesting a role in Fas-mediated apoptosis (Maekawa *et al.*, 1999; Nakai *et al.*, 2000; Ivanov *et al.*, 2003). In addition, microarray analysis identified lymphotoxin B expression upregulated in T-cell senescence. This protein is involved in tumor necrosis factor (TNF) receptor-associated factor-dependent caspase-independent apoptosis suggesting another mechanism by which T-cells may undergo apoptosis during senescence (Force *et al.*, 2000). Furthermore, upregulation of early growth response 2 (EGR-2) may result in apoptosis by two independent mechanisms: (i) increasing permeability of the mitochondrial membrane leading to release of cytochrome c, and (ii) through EGR-2-dependent transcriptional activation of Fas ligand promoter (Unoki & Nakamura, 2003; Yoo & Lee, 2004). Taken together, these data demonstrate that genes involved in apoptosis may be affected in TCC senescence. However, we were unable to detect consistent alterations in caspase-mediated apoptosis or Fas expression detected by fluorescence-activated cell sorting (FACS) analysis (unpublished data) demonstrating the complexity and various mechanisms of regulation of AICD. This area warrants further investigation to determine the contribution of apoptosis, and which type of apoptosis, to TCC dysfunction in aging.

Various genes involved in inflammation were found to be upregulated in TCC senescence, including several CXC chemokines: IL-8, CCL3, CCL2, and CXCL10. IL-8 functions primarily as a chemoattractant and induces secretion of proinflammatory mediators TNF-α, IL-1β, and IL-6. Furthermore, IL-8 is induced by lymphotoxin B receptor, one of the genes that were found to be differentially expressed in TCC senescence. This occurs in an NF-κB and AP-1-dependent manner, further linking these genes and signalling pathways (Chang *et al.*, 2002). In addition to the proinflammatory actions of IL-8, CCL3 is a chemokine that is involved in the acute inflammatory state in the recruitment and activation of polymorphonuclear leukocytes (Wolpe *et al.*, 1988). Another member of the CXC subfamily of cytokines, CCL2, has been implicated in the pathogenesis of diseases characterized by infiltrating monocytes including psoriasis, rheumatoid arthritis and atherosclerosis. CXCL10 is induced by IFN-γ, which is upregulated in inflammation. Binding of CXCL10 to its receptor causes pleiotropic effects, including stimulation of monocytes, natural killer (NK) and T-cell migration and modulation of adhesion molecule expression. This plethora of proinflammatory genes upregulated at immunosenescence is consistent with multiple observations on the low-level inflammatory status commonly found in the elderly, and also associated with poor prognosis in longitudinal studies (Wikby *et al.*, 2006).

Sequence variations, or polymorphisms, in a variety of pro- or anti-inflammatory cytokine genes have been found to influence aging and longevity. Because of its function in regulating both inflammation and immune response, it is hypothesized that transforming growth factor-β1 (TGF-β1) may play a role in aging. In fact a number of polymorphisms within the TGF-β1 promotor have been described and are associated with longevity (Carrieri *et al.*, 2004; Capri *et al.*, 2006). In the current report, we have identified various TGF-β1-regulated genes as differentially expressed in senescent TCCs. These data would suggest that TGF-β1 activity may be altered in senescence and aging, in concordance with previous microarray investigations of myogenic progenitors (Beggs *et al.*, 2004). Furthermore, we have identified several regulators of TGF-β1, including IL-8 and KLF6. Although we have not investigated genetic polymorphisms underlying gene expression changes observed in the current study, it remains possible that genetic variability may contribute to senescence, and warrants further investigation.

One gene that was identified as differentially expressed in TCC senescence, Δ5,6 fatty acid desaturase, may play a major role in the aging process. These enzymes are involved in the metabolism of fatty acids (conversion of linoleic acid to γ-linoleic acid). There is no doubt that the lipid composition of the membrane would influence cellular metabolism and the response to stimuli as it directly modulates the fluidity and as a consequence the interaction of receptors with signalling molecules. It is known that an imbalance of the fatty acid metabolism leads to an inappropriate synthesis of prostaglandin (PG) E2 (Chavali & Forse, 1999). In the current study, we demonstrated that the PG synthase 2 gene is overexpressed while fatty acid desaturase is decreased in TCC senescence (Table 2). Fatty acid desaturase is activated by insulin. However, we found that aging TCCs downregulate the insulin upstream factor 1. It is not known how all these molecules interact during the aging process and we cannot exclude the lipidomic part as a putative contributor to immunosenescence. One possible explanation for the relationship between changes in lipid metabolism and senescence is the influence of membrane lipid on membrane and/or cytosolic protein activation.

Taken together, these data implicate several signalling pathways that may be involved in T-cell senescence, including the TGF-β signalling cascade, which has recently been demonstrated to be important in aging. In addition, it appears that the genes encoding various proinflammatory molecules are upregulated in TCCs as they age and undergo senescence. Furthermore, regulators of fos, EGF, and CTNNB1 (β-catenin) are affected in TCC senescence, suggesting that these pathways may be important contributors to the immunosenescent phenotype. NF-κB is a common link between these signal transduction pathways and its function is regulated by many of the genes we have described in the current report as being differentially expressed in TCC senescence. In addition, altered NF-κB signalling has been described for multiple disease phenotypes. Although we have not specifically investigated NF-κB targets or regulatory elements in this study, the regulation and cooperation of each of these signal transduction pathways in aging and senescence are of great interest and further investigation is necessary to unravel the complex regulatory mechanisms and implications of dysregulated cellular signalling. In particular, it would be interesting to investigate genomic and proteomic changes that occur during the aging process in samples derived from elderly donors, in order to define *in vivo* biomarkers of aging. Proteomic investigations employing Ciphergen-SELDI mass spectroscopy in the same *in vitro* model of immunosenescence under chronic antigenic stress are ongoing.

## Experimental procedures

### Cell culture

Five different CD4^+^ TCCs from the same healthy 85-year-old donor were selected for detailed study here. They were generated from phytohemagglutinin (PHA)-stimulated peripheral blood mononuclear cell (PBMC) by limiting dilution in the presence of IL-2 and pooled irradiated PBMC feeder cells as previously described (Pawelec *et al.*, 2002, 2006b). These five clones were not markedly different in any way from the majority of such clones that we have obtained from numerous different donors, and may therefore said to be ‘representative’. They, and the majority of the other TCCs derived under identical conditions, express markers of effector memory cells and effector cells (CD45RO^++^, CD45RB^+^, CD45RA^lo^, CD28^lo^, CD95^++^, CCR7^lo^), and in addition carry typical markers of activated T-cells (CD80, CD86, PD-L1, MHC class II). They were harvested and cryopreserved at different time points over their finite lifespans. For each assay, the same clone at three to five different time points was tested, ranging from 26 population doublings (PD) up to 66.25 PD, the last time point in all cases representing the time when that particular clone ceased to proliferate (Table 1).

### Microarray procedures

Total RNA was isolated from experimental samples listed in Table 1a using the Trizol reagent (Invitrogen, Carlsbad, CA, USA) and QIAGEN RNeasy kit (QIAGEN, Valencia, CA, USA). RNA from cells of the first time point for each clone was used as the reference against which array RNA from cells of the later time points was analysed. RNA quality was assessed using a Bioanalyzer RNA chip (Agilent, Palo Alto, CA, USA). RNA concentration was quantifed by spectrophotometry and 7 µg were used from each sample. All arrays were carried out in duplicate, the duplicate array being a dye swap.

Approximately 6600 oligonucleotides (20 µm) corresponding to selected human genes involved in cellular signalling, survival, immune response, inflammation, proliferation/differentiation, and immune response were purchased from Sigma-Genosys Ltd (Pampisford, UK) and arrayed onto Codelink activated microarray slides (Amersham Biosciences, Piscataway, NJ, USA) using a Biorobotics MicroGrid arrayer (Genomic Solutions, Ann Arbor, MI, USA). A 32-pin spotting configuration was used to print a batch of slides centrally within the slide's active surface. Within the design each oligonucleotide was replicated on each array to provide greater statistical measurement for each gene. In addition, also incorporated within the array is a set of 10 standard spike control oligonucleotides, purchased from Stratagene (La Jolla, CA, USA), to assess quality of the hybridization, negative controls, spotting buffer controls, and landing lights (Cy3 prelabelled oligonucleotide) to define block parameters. Printing conditions included a constant temperature of 20 °C and constant humidity of 45% as suggested by the manufacturers. Amine coupling of the oligonucleotides to the slide surface and post processing of the slides was performed according to the manufacturer's protocol (Amersham Biosciences). Labelling was performed using the Genisphere 3DNA labelling kit according to the manufacturer's protocol (Genisphere Inc., Hatfield, PA, USA).

Prior to hybridization the purified, labelled samples were combined into a formamide-based hybridization mix containing 4% (w/v) dextran sulphate. Hybridization was performed using an optimized protocol for the Lucidea SlidePro hybridization station (Amersham). Commencement of hybridization was within 1 h of the completion of the slide preprocessing. The initial cDNA hybridization was performed for 17 h at 42 °C with gentle mixing, washed and dried. The subsequent dendrimer hybridization was performed for 4 h at 50 °C with gentle mixing in the same formamide-based hybridization solution with the exception that the concentration of dextran sulphate was reduced to 2.5% (w/v). Microarrays were stored in the dark until scanning was performed using a GSI Luminomics 4000XL scanner using the manufacturer's software (Scanarray, version 3.0). Both Cy3 (546) and Cy5 (646) channels were scanned separately using laser power and PMT settings optimized for high signal : noise ratios and balanced channel outputs. Individual .tif images generated for each channel for each array were then saved for subsequent comparison. Numerical values were extracted from the combined two channel .tif images obtained from the Scanarray software using GenePix 4.1 (Molecular Devices Corporation, Sunnyvale, CA, USA). Following extraction, the data generated were saved as an excel file for subsequent use within GeneSpring expression analysis software (Silicon Genetics, Redwood City, CA, USA) and are included in Supplementary Table S1.

### Statistical analysis of data

Data files were saved as text tab-delimited files, copied, and saved into the same folder. Data were combined into a ‘new experiment’, to which loess and dye-swap normalizations were applied. Control spots were removed and loess normalization applied. The cross-gene error model was switched off and the data analysed twice (the second time with channels reversed for all chips) in order to adjust for the inherent ‘channel-bias’ present in GeneSpring. GeneSpring software was used to generate the principle components analysis (PCA) profiles. A subset of genes for data interrogation was generated that excluded controls, any genes that did not dye swap, any spots of poor quality, and genes expressed below the detection threshold. For these selected genes, data were filtered on fold change. A second analysis, filtering on confidence, was possible because genes were represented on the chip by multiple spots. Analysis of variance and parametric tests were used to investigate the effect of time in culture on gene expression. Benjamini and Hochberg multiple correlations analysis was controlled by applying the false discovery rate (FDR) algorithm at FDR < 0.1 (*P <* 0.05).

### Pathway analysis

A total of 43 genes that had a FDR < 0.1 in at least three of the five clones investigated were used for pathway analysis. GENBANK accession numbers were imported into and mapped to the Ingenuity database using Ingenuity Pathway Analysis software (Ingenuity Systems, Redwood City, CA, USA). The identified genes were mapped to biological networks available in the Ingenuity database and were then ranked by score. The score is the probability that a group of genes equal to or greater than the number in a network could be achieved by chance alone. A score of 3 or higher equates to a 99.9% confidence of not being generated by chance alone, and was used as a cutoff for significance.

### Real-time quantitative reverse transcription-polymerase chain reaction

The genes and TaqMan (Applied Biosystems, Applera, UK) probes used for RT-PCR are listed in Table 3*.* RNA was prepared from the TCCs listed in Table 1b and *in vitro* transcription was performed with 1 µg total RNA using random hexamer primers (Invitrogen). The Bio-Rad iCycler (Bio-Rad, Hercules, CA, USA) with FAM-490 system detection was used for real time RT-PCR. PCR thermocycler conditions were 50 °C for 2 min, 90 °C for 2 min followed by 45 cycles of 95 °C for 15 s and 60 °C for 60 s. Six TCCs isolated from three different octogenarian donors were run in triplicate with both test probes and the control gene human GAPDH to control for differences in amount of starting material. A standard curve was created for each PCR. Fold changes were calculated by normalizing the test crossing threshold (Ct) with the GAPDH amplified control Ct.

## Supplementary material

The following supplementary material is available for this article:

Table S1 Extracted data used for GeneSpring analysis of T-cell clone senescence.

This material is available as part of the online article from:

http://www.blackwell-synergy.com/doi/abs/10.1111/j.1474-9726.2007.00269.x (This link will take you to the article abstract).
